# Extracorporeal adsorption of protective and toxic bile acids and bilirubin in patients with cholestatic liver dysfunction: a prospective study

**DOI:** 10.1186/s13613-023-01198-7

**Published:** 2023-11-09

**Authors:** Antonia Greimel, Katharina Habler, Caroline Gräfe, Nils Maciuga, Clara Isabell Brozat, Michael Vogeser, Michael Zoller, Felix L. Happich, Uwe Liebchen, Sandra Frank, Michael Paal, Christina Scharf

**Affiliations:** 1 Department of Anesthesiology, LMU Hospital, Marchioninistrasse 15, 81377 Munich, Germany; 2 Institute of Laboratory Medicine, LMU Hospital, Munich, Germany

**Keywords:** Bile acids, Cholestasis, Critically ill patients, Cytosorb^®^, Cholestatic liver failure, Mass spectrometry

## Abstract

**Background:**

The release of toxic bile acids (BAs) in the blood of critically ill patients with cholestatic liver dysfunction might lead to the damage of various organs. Their extracorporeal elimination using the cytokine adsorber Cytosorb^®^ (CS) (adsorption of especially hydrophobic molecules < 60 kDa) might be promising, but data proving a potential adsorption are missing so far.

**Methods:**

The prospective Cyto-SOVLE study (NCT04913298) included 20 intensive care patients with cholestatic liver dysfunction, continuous kidney replacement therapy, total bilirubin concentration > 10 mg/dl and the application of CS into the dialysis circuit. Bilirubin and different BAs were measured pre- and post-CS at defined timepoints (10 min, 1, 3, 6, and 12 h after initiation). Relative reduction (RR, %) was calculated with: $1-\left(\frac{\mathrm{concentration} (\mathrm{pre}-\mathrm{post})}{\mathrm{concentration} (\mathrm{pre})}\right)*100$.

**Results:**

The median RR for total and conjugated bilirubin after initiation was − 31.8% and − 30.3%, respectively, and decreased to − 4.5% and − 4.8% after 6 h. A high initial RR was observed for the toxic BAs GCA (− 97.4%), TCA (− 94.9%), GCDCA (− 82.5%), and TCDCA (− 86.0%), decreasing after 6 h to − 32.9%, − 32.7%, − 12.8%, and − 14.3%, respectively. The protective hydrophilic BAs showed a comparable RR after initiation (UDCA: − 77.7%, GUDCA: − 83.0%, TUDCA: − 91.3%) dropping after 6 h to − 7.4%, − 8.5%, and − 12.5%, respectively.

**Conclusions:**

Cytosorb^®^ can adsorb bilirubin and toxic as well as protective BAs. However, a fast saturation of the adsorber resulting in a rapid decrease of the RR was observed. Furthermore, no relevant difference between hydrophobic toxic and hydrophilic protective BAs was detected regarding the adsorption amount. The clinical benefit or harm of the BA adsorption needs to be evaluated in the future.

**Supplementary Information:**

The online version contains supplementary material available at 10.1186/s13613-023-01198-7.

## Introduction

While primary acute liver failure is a rare clinical entity in critical care, secondary acquired liver dysfunction is commonly found in critically ill intensive care unit (ICU) patients [1]. The causes vary, whereby hypoxia, inflammation, or toxic side effects are the most common triggers [2]. The manifestation may be as primary cholestatic or hypoxemic liver function impairment that both have an incidence of 10–30% in the critically ill [3, 4]. To date, there is no unified definition of secondary acquired liver dysfunction. However, cholestatic liver failure primarily manifests by an increase of bile acids (BA), conjugated bilirubin, and liver enzymes, such as alkaline phosphatase (AP), and γ-glutamyltransferase (γ-GT) [3, 5]. The mortality of the aforementioned condition has been reported to be as high as 50%, so effective treatment would be desirable [6].

The elevation of bilirubin in ICU patients is associated with poor outcome, but bilirubin itself has no direct toxic side effects (e.g., brain oedema) in adult patients [7]. In contrast, several circulating BAs have direct toxic effects. The metabolism of BAs is complex and not completely understood to date. They are subject to enterohepatic circulation and there is no relevant release into the blood in healthy adults [8]. Patients with cholestasis have a retention of BAs in the liver with a subsequent discharge into the blood and other tissues [9]. BAs can be divided into protective and toxic BAs, with the protective ones being more hydrophilic and the toxic ones more hydrophobic. Toxic BAs include taurocholic acid (TCA), taurochenodeoxycholic acid (TCDCA), glycocholic acid (GCA) and glycochenodeoxycholic acid (GCDCA). Their accumulation potentially lead to a permanent damage of various organs, such as the liver [10], lung [11], heart [12], and kidney [13].

In contrast, there are protective BAs, including ursodeoxycholic acid (UDCA) and its secondary glycine- (GUDCA) and taurine-conjugated (TUDCA) forms. In particular, UDCA is used as a therapeutic strategy in the context of cholestatic liver failure to ultimately minimize toxic side effects [14]. Another potential approach to eliminate BAs might be the use of the cytokine adsorber Cytosorb^®^ (CS) that is approved for the elimination of bilirubin. CS is a cartridge that can be integrated into an extracorporeal circuit (mostly continuous dialysis). It contains small highly porous polysterol beads with an adsorption surface area of > 45,000 m^2^. The adsorption spectrum of CS includes primarily hydrophobic substances with a molecular size of about < 60 kDa. A reduction of bilirubin in the blood using CS has already been demonstrated [15]. Furthermore, the elimination of especially hydrophobic toxic BAs seems, based on mechanistical considerations, possible. Until now, only two case reports demonstrated a decrease of total BAs in patients´ blood after its usage [16, 17].

To date, the adsorption performance of the cartridge is neither known for bilirubin nor for the various BAs. Furthermore, it has not been investigated whether the cartridge is saturated during the course of therapy. The question of interest was, therefore, to evaluate the adsorption performance and saturation kinetics of CS for bilirubin and various toxic and protective BAs determined by mass spectrometry in intensive care patients with cholestatic liver failure.

## Methods

### Study setting

This was a monocentric, prospective study investigating the adsorption rate and saturation kinetics of CS for bilirubin and various BAs in patients with cholestatic liver dysfunction. Patients were included between May 2021 and August 2022 during their stay at two ICUs at the LMU hospital in Munich. The local institutional review board approved the study (registration number 21-0236). The study was registered at clinical trials (NCT04913298). Written informed consent was obtained from the patients or their legal representatives in line with the vote of the review board prior to study inclusion. The study protocol conforms to the ethical guidelines of the 1975 Declaration of Helsinki as reflected in a priori approval by the institution’s human research committee.

### Study population

The study included adult patients (≥ 18 years) with the necessity of continuous kidney replacement therapy (CKRT) mostly due to an acute kidney injury (AKI) stage 2 or 3 diagnosed by the AKI classification of the KDIGO consensus criteria (see Table 2) [18]. The indication of starting CKRT and the modality (CVVHD or CVVHDF) was at the responsibility of the attending physician; reasons can be found in Table 2. Furthermore, total bilirubin in patients’ blood had to be > 10 mg/dl prior to CS application. Exclusion criteria were no consent to the participation in the study and prior CS application.

### Blood sampling

CS was installed post-dialyzer into the Fresenius MultiFiltrate circuit (MultiFiltrate Ultraflux^®^ AV 1000S). Blood samples (EDTA tubes) were taken at the extracorporeal circuit directly before the cartridge (= pre-CS) and directly after the cartridge (= post-CS) at specified timepoints. Sampling timepoints were 10 min after starting CS treatment, and 1, 3, 6, and 12 h after initiation. Different bile acids were also measured in the arterial blood shortly before initiation of CS, after 6, and after 12 h. EDTA anticoagulated plasma was obtained by centrifugation of whole blood at the ICU immediately after sampling. Separated plasma samples were immediately frozen and stored stably at − 80 °C until measurement within 6 months.

### Data collection

For data evaluation, demographic data and clinical and laboratory variables were collected from the electronic laboratory and patient information system. Different laboratory parameters were measured shortly before CS initiation in the clinical routine.

### Laboratory measurements

Total and direct bilirubin were quantified with the diazo reaction on the Cobas^®^ 8000 c702 modular clinical chemistry analyzer (Roche Diagnostics, Mannheim, Germany). BA profiling was done by isotope dilution LC–MS/MS with the Biocrates^®^ Bile Acids Kit (Biocrates, Innsbruck, Austria) on an Acquity ultra-high-performance liquid chromatography system interconnected with a triple quadrupole mass spectrometer Xevo TQ-S (Waters, Milford, MA, USA). Briefly, 10 µL internal standards mix solution was first added to each well of the 96 well plate with exception of the blank well position and dried for 5 min with a nitrogen evaporator. Thereafter, 15 µL of blank, calibration standard, quality control, or patient sample were added to the wells and the plate was dried for 20 min under nitrogen. The analytes were extracted by the addition of 100 µL methanol to each well by shaking for 20 min at 600 rpm and were collected in a capture plate by centrifugation for 2 min at 500 g. Finally 60 µL MilliQ water were added to each well of the capture plate, briefly mixed for 5 min at 450 rpm and the plate was loaded into an autosampler (8 °C sample cooling). The injection volume was 20 µL. CS treatment series in which BA concentrations (e.g., for GUDCA) exceeded the highest calibrator were diluted with phosphate-buffered saline pH 7.4 and concentrations calculated back.

### Statistical analysis

Statistical analysis was performed with IBM SPSS statistics (Version 29.0. IBM Corp., Armonk, NY, USA). *T* test with associated samples was used to compare the concentrations pre- and post-CS after examination of a normal distribution of the examined parameters (Shapiro–Wilk test). Relative reduction (RR) of the parameters by CS at different timepoints was calculated with:

$$\mathrm{Relative}\,\mathrm{reduction} \left(\%\right)=1-\left(\frac{\mathrm{concentration} (\mathrm{pre}-\mathrm{post})}{\mathrm{concentration} (\mathrm{pre})}\right)*100$$

In addition, the BA and bilirubin clearances of CS were calculated with:

$$\mathrm{Clearance} \left(\frac{\mathrm{ml}}{\mathrm{min}}\right)=\mathrm{bloodflow}*\frac{\mathrm{concentration} (\mathrm{pre}-\mathrm{post})}{\mathrm{concentration} (\mathrm{pre})}$$

## Results

### Demographic and clinical data

In total, 20 patients were included in the evaluation. All patients had a cholestatic liver injury and were treated with UDCA with a daily dose of 1 g. The intended duration of CS therapy was 12 h, which was possible in 16 patients. The extracorporeal circuit clotted in four patients between 6 and 12 h, so the last data point after 12 h is missing in those four patients. The median age was 53 years and 70% were male. The SAPS II on the day of the CS treatment was 78 points and the 28-day mortality was 45%. The reasons for the admission at the ICU were in descending order: solid organ transplantation (lung or liver, 30%), acute respiratory distress syndrome (ARDS, 25%), cardiogenic shock or acute-on-chronic liver failure (each 15%), septic shock (10%), and pulmonary artery embolism (5%). Detailed patient characteristics and laboratory parameters measured immediately before initiation of CS can be found in Table 1. Table 2 illustrates details about the kidney replacement therapy.

**Table 1:** Patient characteristics and laboratory measurements

	*n* (%) or median [IQR]
Patient characteristics	
Age (years)	53 [37, 63]
Gender: male/female	14 (70)/6 (30)
Weight (kg)	79 [64, 85]
Height (m)	1.69 [1.65, 1.80]
28-day mortality	9 (45)
SAPS II	78 [69, 90]
Norepinephrine n (%), and dosage (mg/h) at start	17 (85), 0.90 [0.60, 1.60]
Norepinephrine n (%), and dosage (mg/h) after 12 h	12 (60), 0.65 [0.50, 1.20]
Vasopressin n (%), and dosage (IE/h) at start	7 (35) 2.0 [1.5, 2.5]
Vasopressin n (%), and dosage (IE/h) after 12 h	5 (25) 2.0 [0.5, 2.0]
Laboratory parameters before initiation of Cytosorb^®^	
Total bilirubin (mg/dl)	14.7 [11.6, 18.6]
Conjugated bilirubin (mg/dl)	12.5 [10.0, 15.1]
Total BAs (µmol/l)	59 [44, 140]
Alanine aminotransferase (U/l)	160 [96, 271]
Aspartate aminotransferase (U/l)	180 [115, 677]
γ-Glutamyltransferase (U/l)	446 [96, 941]
Alkaline phosphatase (U/l)	368 [160, 780]

*SAPS II* Simplified Acute Physiology Score, *BA* bile acid

**Table 2:** Kidney replacement therapy

Reasons for CKRT	
AKI stage 2 (KDIGO)	6 (30)
AKI stage 3 (KDIGO)	11 (55)
Hyperkalemia	2 (10)
Lactate acidosis	1 (5)
Dialyzer	Fresenius MultiFiltrate circuit (MultiFiltrate Ultraflux® AV 1000S)
CVVHD (CiCa^®^)/CVVHDF (MultiBic^®^, post-dilution)	15 (75)/5 (25)
Clotting	2 (13.3)/2 (40)
Blood flow (ml/min)	100 [100, 162]
CVVHD	100 [100, 100]
CVVHDF	200 [200, 300]
Dialysate flow (ml/h)	2000 [2000, 2125]
Substitute flow (if CVVHDF) (ml/h)	2000 [1000, 3500]
Anticoagulation	
Citrate	15 (75)
Unfractioned Heparin < 1000 IE/h	8 (40)
Unfractioned Heparin > 1000 IE/h	4 (20)
Agatrobane	5 (25)
None	3 (15)

*CKRT* continuous kidney replacement therapy, *CVVHD(F)* continuous veno-venous hemodialysis/hemodiafiltration

### Relative reduction of bilirubin and different bile acids

#### Bilirubin

There was a significant (*p* < 0.01) reduction of total and conjugated bilirubin post- vs. pre-CS at all timepoints. The median RR of total and conjugated bilirubin after 10 min was − 31.8% and − 30.3%, respectively. It decreased rapidly to − 4.5% and − 4.8% after 6 h of CS application. The results of all measured bilirubin concentrations can be found in Additional file 1: Table S1a and Table S1b. Additional file 1: Figure S1 displays the relative reduction (%) of bilirubin during CS application as boxplots. Table 3 illustrates the clearance of bilirubin and bile acids by Cytosorb^®^ during application.

**Table 3:** Clearance (ml/min) of bilirubin and bile acids by Cytosorb^®^

	10 min	1 h	3 h	6 h	12 h
Total bilirubin median (IQR)	35.2 (31.1, 41.3)	13.6 (10.7, 16.4)	7.6 (7.1, 10.3)	6.3 (4.6, 7.3)	3.1 (2.1, 4.0)
Conjugated bilirubin median (IQR)	34.8 (30.6, 41.3)	12.9 (10.8, 15.2)	7.4 (5.2, 9.8)	6.1 (5.0, 7.4)	2.9 (1.4, 5.1)
GCA median (IQR)	99.5 (98.1, 103.8)	84.9 (76.2, 91.6)	59.3 (50.9, 70.3)	38.7 (32.7, 52.2)	21.3 (16.1, 40.0)
TCA median (IQR)	97.0 (95.5, 103.0)	78.4 (71.2, 87.4)	52.7 (43.5, 67.2)	38.4 (30.8, 46.7)	17.7 (10.2, 34.7)
GCDCA median (IQR)	85.3 (81.6, 89.0)	37.9 (20.0, 42.7)	18.1 (12.9, 26.0)	17.3 (9.4, 21.5)	8.1 (3.4, 16.7)
TCDCA median (IQR)	88.8 (79.9, 93.7)	37.3 (27.4, 43.2)	24.6 (16.0, 28.2)	15.5 (12.1, 18.9)	11.9 (5.9, 21.0)
UDCA median (IQR)	80.4 (77.7, 88.4)	37.4 (28.5, 48.8)	18.2 (14.4, 23.8)	10.3 (1.2, 27.0)	− 1.5 (− 6.6, 14.7)
GUDCA median (IQR)	91.7 (77.1, 93.7)	39.8 (26.9, 48.0)	22.7 (12.0, 31.2)	12.9 (7.2, 15.7)	4.5 (− 1.3, 14.6)
TUDCA median (IQR)	94.8 (86.7, 97.9)	50.6 (23.4, 58.6)	28.8 (19.5, 35.2)	17.0 (10.9, 24.9)	6.6 (− 1.6, 16.4)

*GCA* glycocholic acid, *TCA* taurocholic acid, *GCDCA* glycochenodeoxycholic acid, *TCDCA* taurochenodeoxycholic acid, *UDCA* ursodeoxycholic acid, *GUDCA* glycoursodeoxycholic acid, *TUDCA* tauroursodeoxycholic acid

#### Toxic bile acids

No patient showed an increase of the primary BA cholic acid (CA) during CS treatment and the measured concentrations were mostly (90%) under the lower limit of quantification. However, the conjugated forms were commonly increased. There was a significant (*p* < 0.01) reduction of glycocholic acid (GCA) and taurocholic acid (TCA) post- vs. pre-CS at all timepoints. The median RR of GCA and TCA after initiation was − 97.4% and − 94.9%, which dropped to − 32.9% and − 32.7% after 6 h, respectively.

**Fig. 1:**
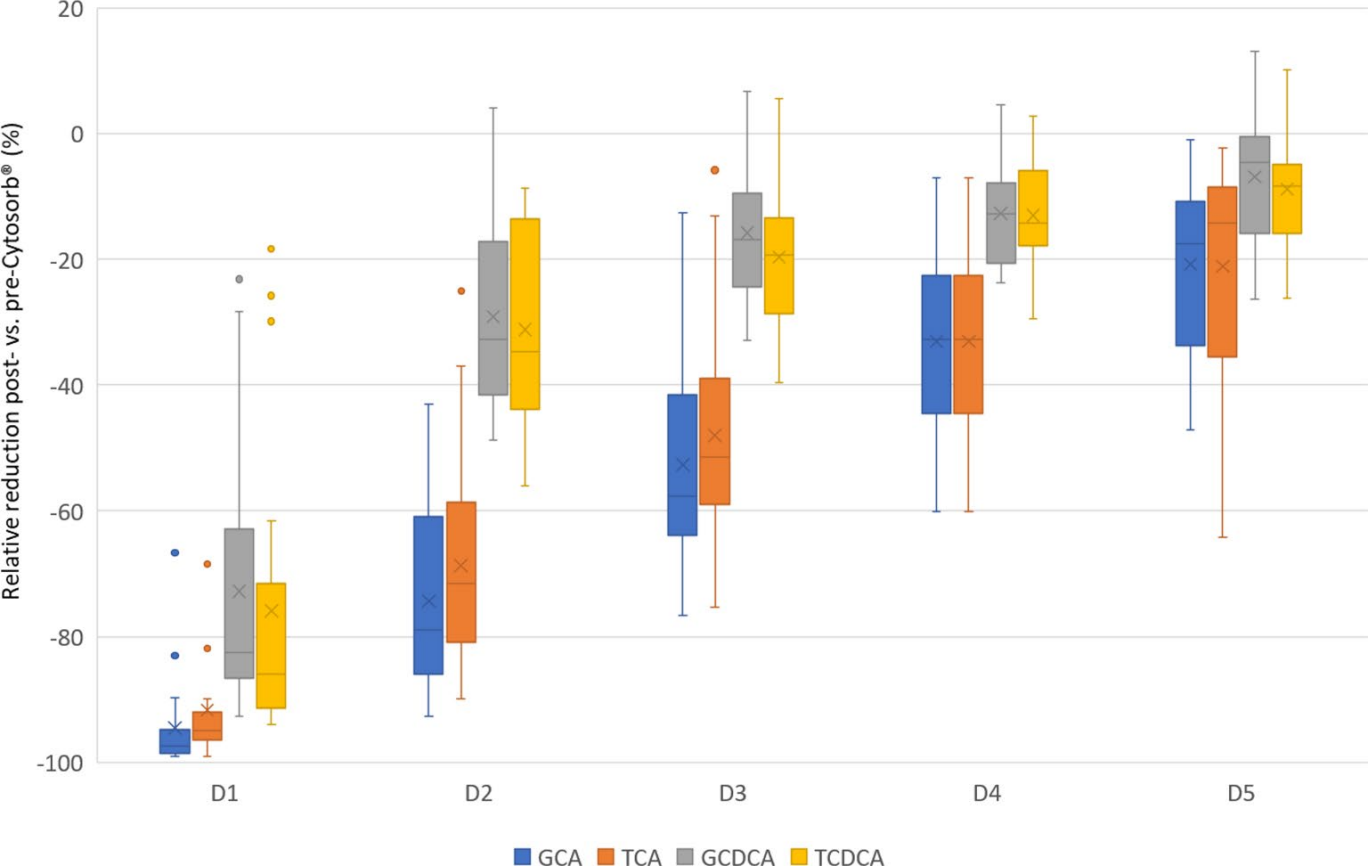
Relative reduction (%) of toxic bile acids with Cytosorb^®^. D1: 10 min after initiation of Cytosorb^®^, D2: 1 h after initiation of Cytosorb^®^, D3: 3 h after initiation of Cytosorb^®^, D4: 6 h after initiation of Cytosorb^®^, D5: 12 h after initiation of Cytosorb^®^, blue boxplots represent the relative reduction of GCA, orange ones of TCA, grey ones of GCDCA, and yellow ones of TCDCA. The boxes of the boxplots represent the interquartile range (IQR) and the line the median. Whiskers were limited to 1.5 times the IQR. The cross represents the mean

The concentrations of the second primary BA chenodeoxycholic acid (CDCA) were also mostly under the lower limit of quantification, whereas the conjugated forms were often increased. There was a significant (*p* < 0.03) extracorporeal reduction of glycochenodeoxycholic acid (GCDCA) and taurochenodeoxycholic acid (TCDCA) at all timepoints. The median RR of GCDCA and TCDCA after initiation was − 82.5% and − 86.0%, which dropped to − 12.8% and − 14.3% after 6 h, respectively. The results of all toxic BA concentrations can be found in Additional file 1: Table S2a–d. Figure 1 illustrates the relative reduction (%) of all toxic BAs during therapy as boxplots.

#### Protective bile acids

**Fig. 2:**
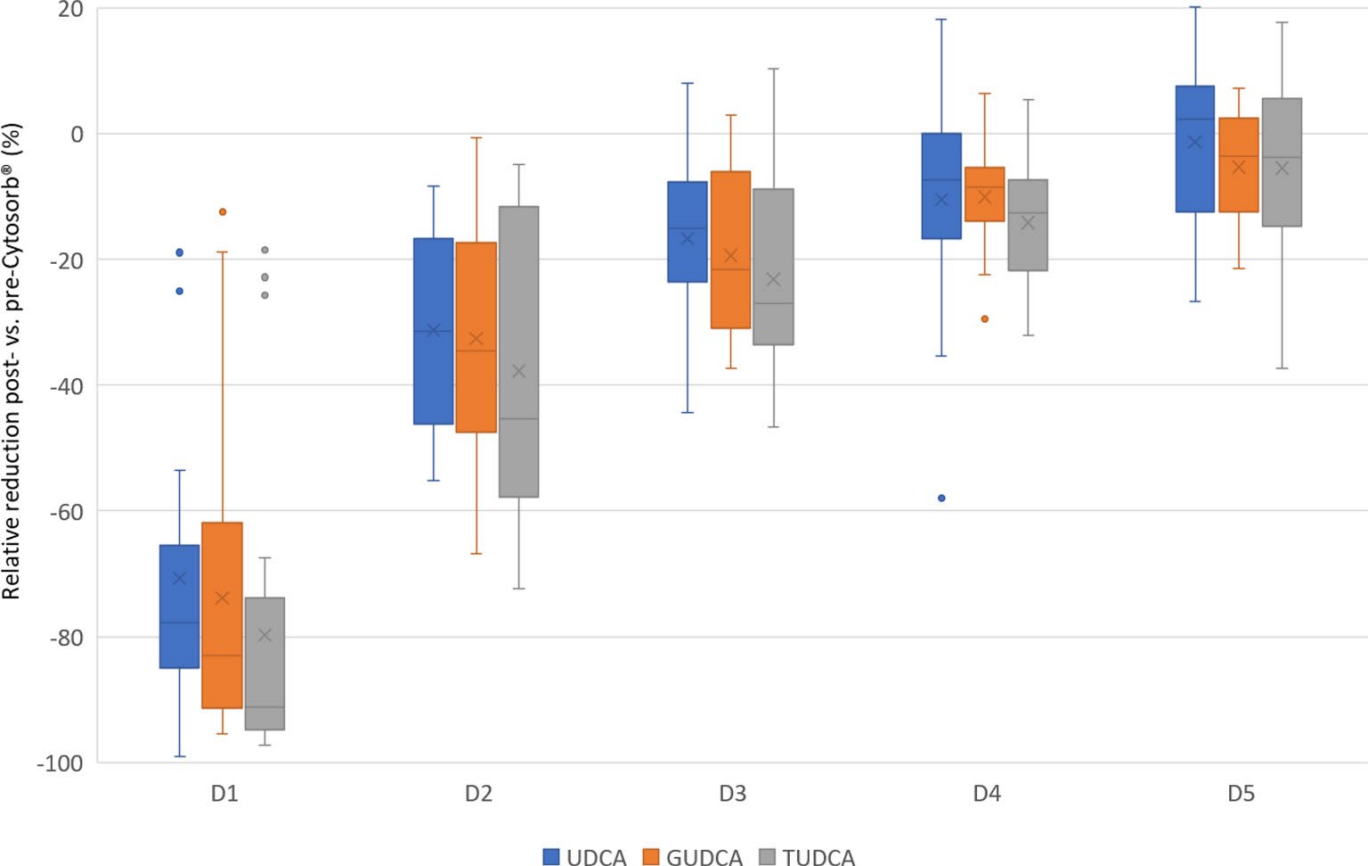
Relative reduction (%) of protective bile acids with Cytosorb^®^. D1: 10 min after initiation of Cytosorb^®^, D2: 1 h after initiation of Cytosorb^®^, D3: 3 h after initiation of Cytosorb^®^, D4: 6 h after initiation of Cytosorb^®^, D5: 12 h after initiation of Cytosorb^®^, blue boxplots represent the relative reduction of UDCA, orange ones of GUDCA, and grey ones of TUDCA. The boxes of the boxplots represent the interquartile range (IQR) and the line the median. Whiskers were limited to 1.5 times the IQR. The cross represents the mean

The secondary bile acids lithocholic acid (LCA) and deoxycholic acid (DCA) were mostly under the lower limit of quantification. Most patients had increased blood concentrations of the secondary BA ursodeoxycholic acid (UDCA). There was a significant (*p* < 0.01) reduction of UDCA post- vs. pre-CS at 10 min, 1 h, and 6 h. No significant reduction was observed after 3 (*p* = 0.07) and 12 h (*p* = 0.25). The median RR after 10 min was − 77.7% that decreased to − 7.4% after 6 h. Similar RRs were observed for the glycine-conjugated (GUDCA) and taurine-conjugated (TUDCA) forms. There was a significant (*p* < 0.01) reduction of GUDCA and TUDCA at 10 min, 1, 3, and 6 h. No significant reduction was observed after 12 h (GUDCA: *p* = 0.35, TUDCA: *p* = 0.15). The median RR for GUDCA and TUDCA after 10 min were − 83.0% and − 91.3% both dropping to − 8.5 and − 12.5 after 6 h. The results of all protective BA concentrations can be found in Additional file 1: Table S3a–c. Figure 2 illustrates the relative reduction (%) of all protective BAs during therapy as boxplots.

### Clearance of bilirubin and different bile acids by Cytosorb^®^

The median clearance of total and conjugated bilirubin by CS was 35.2 and 34.8 ml/min after 10 min, representation about one-third of the blood flow of the extracorporeal circuit. The clearance dropped markedly to about 6 ml/min after 6 h for both total and conjugated bilirubin.

The median clearance of GCA and TCA after CS initiation was 99.5 and 97.0 ml/min (about 98% of the blood flow). The clearance decreased fast to 38.7 and 38.4 ml/min after 6 h. Slightly lower clearances were observed for GCDCA and TCDCA. The median clearance was after initiation 85.3 and 88.8 ml/min, dropping to 17.3 and 15.5 ml/min after 6 h, respectively.

**Fig. 3:**
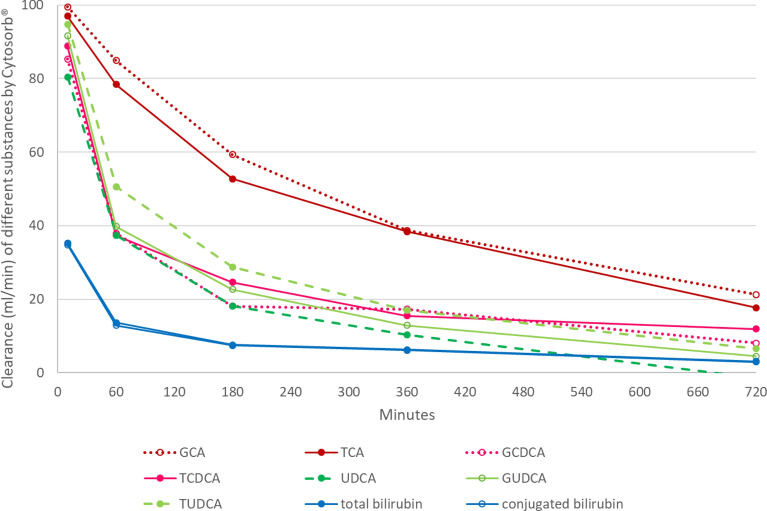
Median clearance of different bile acids and total and conjugated bilirubin with Cytosorb^®^ during the course of therapy. *GCA* glycocholic acid, *TCA* taurocholic acid, *GCDCA* glycochenodeoxycholic acid, *TCDCA* taurochenodeoxycholic acid, *UDCA* ursodeoxycholic acid, *GUDCA* glycoursodeoxycholic acid, *TUDCA* tauroursodeoxycholic acid

The median UDCA clearance by CS was 80.4 ml/min after 10 min and 10.3 ml/min after 6 h. The median clearances of GUDCA and TUDCA after initiation were 91.7 and 94.8 ml/min, respectively. They dropped to 12.9 and 17.0 ml/min after 6 h. Figure 3 illustrates the median clearances (ml/min) of all bile acids and total and conjugated bilirubin during the course of Cytosorb^®^ application.

### Change of different bile acids and bilirubin during the application of Cytosorb^®^

**Fig. 4:**
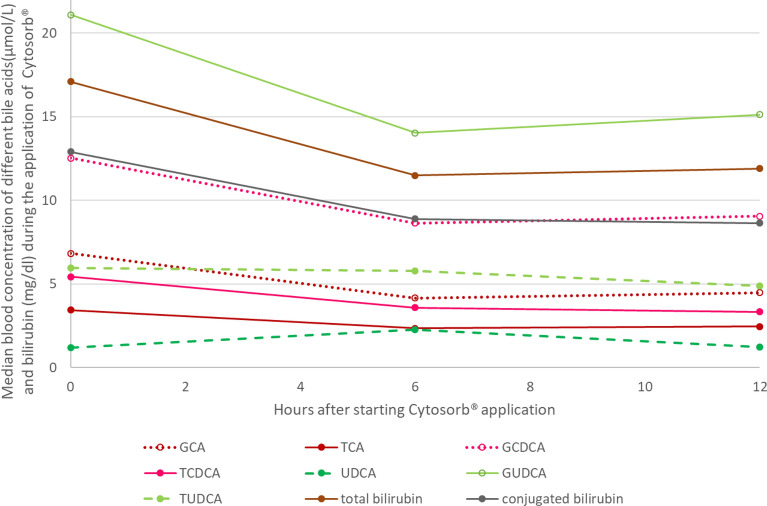
Median blood concentration of different bile acids and bilirubin during CS application. *GCA* glycocholic acid, *TCA* taurocholic acid, *GCDCA* glycochenodeoxycholic acid, *TCDCA* taurochenodeoxycholic acid, *UDCA* ursodeoxycholic acid, *GUDCA* glycoursodeoxycholic acid, *TUDCA* tauroursodeoxycholic acid

The change of different bile acids and bilirubin in the blood was evaluated in patients with the full treatment interval (*n* = 16). There was a significant (*p* < 0.01) reduction of conjugated and total bilirubin as well as all bile acids except UDCS between start and 6 h of treatment. No significant reduction was observed for any parameter between 6 and 12 h. The blood concentrations of the different bile acids can be found in Additional file 1: Table S4. The change of the concentration during therapy can be found in Fig. 4.

## Discussion

Severe hepatic failure is one of the most dangerous diseases in critically ill patients and extracorporeal liver support devices are time- and cost-intensive without extensive evidence for improved outcomes [19, 20]. The cytokine adsorber Cytosorb^®^ has a CE-mark for the elimination of bilirubin and can, therefore, be used for patients with liver dysfunction. As it is easy to handle, its use might be promising in those patients [15]. However, in-vivo data about the elimination rate and capacity are lacking for bilirubin as well as for the more important bile acids.

Basically, it is important to know, which molecules can theoretically be eliminated by CS based on their molecular size. The cutoff value of CS based on manufacturer’s specifications is about up to 60 kDa. Poli et al. described various substances that might be adsorbed by CS [21], but recent in-vivo data studies showed no adsorption of small hydrophilic molecules (i.e., meropenem or ammonia) which are named in this article [22, 23]. An adsorption of bilirubin and most bile acids might be possible due to their molecular weight and their lipophilicity and was previously demonstrated in-vitro [24]. Our study provides an important background for or against the use of CS in patients with cholestatic liver failure.

All patients showed an initial high relative reduction of four rather hydrophobic toxic and three rather hydrophilic protective bile acids by CS with median adsorption rates > 70%. Though, there was a rapid decrease in RR during the procedure, indicating a brisk saturation of the adsorber. In addition, positive relative reduction rates were observed during the course of application; i.e., higher concentrations post-CS than pre-CS. Since there are only small deviations, it could be caused by the measurement. Another possibility is the release of substances from the cartridge into the blood during the application. This cannot be conclusively clarified within the scope of the study. The initial high clearance of BAs resulted in a significant decrease of toxic bile acids in the blood during the first 6 h of treatment. In contrast, no change was then perceived between 6 and 12 h. Most likely, no decrease in protective bile acids in patients’ blood were observed due to the administration of UDCA several times a day. The relative reduction of bilirubin was significantly lower at 30%. However, removal of BAs is more relevant than bilirubin in adult patients due to their toxic potential [25, 26]. Furthermore, a high clotting rate of in total 20% between 6 and 12 h of treatment was observed. The particularly high rate of clotting events in patients treated with CVVHDF (40%) should be noted regarding its safe use as well.

Wallon et al. showed a decrease in total BAs in the blood of critically ill patients using two albumin dialysis systems [27] and a recent work demonstrated a reduction of BAs in the blood of about 30% by the application of coupled plasma filtration and adsorption [28]. However, the measurement of total BAs is questionable, since it remains unclear which ones (hydrophobic vs. hydrophilic) were primarily removed. For example, UDCA is regularly used for the treatment of cholestatic liver dysfunction in ICU patients [7]. Recent data exemplify that transplant free survival in patients with secondary sclerosing cholangitis can be increased by the use of UDCA [29]. Knowledge of the adsorption of UDCA by CS is important and strategies should be developed to avoid negative effects due to UDCA removal by CS (e.g., additional administration during CS application). In addition, it must be questioned and investigated in the future whether the benefit of eliminating toxic bile acids outweighs the potential harm concerning the elimination of protective bile acids.

There were large inter-patient differences in the concentration and distribution of bile acids in the blood. Therefore, the indication for the use of CS should not be based on the total bilirubin concentration or the amount of total BAs in the blood. In the authors' view, the application of CS should be determined primarily by the concentration of toxic BAs in the blood. In the future, outcome-based studies should investigate a benefit or harm of CS application during cholestatic liver failure. Not only focusing on the reduction of bilirubin or total BAs [30], but rather on valid endpoints such as mortality or an increase in liver function would be desirable.

Finally, this study has several limitations. As the focus of this study lied on the elimination kinetics with extracorporeal measurement of different substances, no statement can be made on the change in patients´ outcome by the use of CS. Furthermore, both CVVHD and CVVHDF were used as dialysis modalities; however, as bilirubin and bile acids were directly measured pre- and post-CS, this should not have any influence on the clearance by CS. However, we cannot exclude whether a higher blood flow leads to an even faster saturation of the adsorber. Since the study only investigated the clearances by CS after the blood has passed the dialysis membrane, the dialyzer might remove some soluble substances (e.g., bilirubin). The additional clearance by the dialysis membrane cannot be estimated in this setting. Finally, some of the measured BAs were below the lower limit of quantification in the blood in most of the patients. No statement can be made about the potential adsorption of these BAs.

## Conclusion

The cytokine adsorber Cytosorb^®^ can remove bilirubin and various bile acids in critically ill patients with cholestatic liver dysfunction. However, a fast decrease of the reduction rate indicates a brisk saturation of the cartridge, resulting in a reduced effectiveness. Furthermore, no relevant difference was observed in the adsorption of hydrophobic and hydrophilic BAs, thus primary protective BAs were also removed. Studies that focus on differences in patients’ outcome should follow in the future.

### Supplementary Information

**Additional file 1.** **Table S1a:** Total bilirubin concentration (mg/dL) pre- and post-Cytosorb® at different timepoints. **Table S1b:** Conjugated bilirubin concentration (mg/dL) pre- and post-Cytosorb® at different timepoints. **Table S2a:** GCA (µmol/L) concentration pre- and post-Cytosorb® at different timepoints. **Table S2b:** TCA (µmol/L) concentration pre- and post-Cytosorb® at different timepoints. **Table S2c:** GCDCA (µmol/L) concentration pre- and post-Cytosorb® at different timepoints. **Table S2d:** TCDCA (µmol/L) concentration pre- and post-Cytosorb® at different timepoints. **Table S3a:** UDCA (µmol/L) concentration pre- and post-Cytosorb® at different timepoints. **Table S3b:** GUDCA (µmol/L) concentration pre- and post-Cytosorb® at different timepoints. **Table S3c:** TUDCA (µmol/L) concentration pre- and post-Cytosorb® at different timepoints. **Table S4:** Blood concentration of different bile acids (µmol/L) before the start of Cytosorb® and after six and twelve hours after initiation. **Figure S1:** Relative reduction (%) of total bilirubin and conjugated bilirubin at different timepoints.

## Data Availability

All data generated during this study are included in this article.

## Abbreviations

AKI: Acute kidney injury
AP: Alkaline phosphatase
APACHE: Acute physiology and chronic health evaluation
BA: Bile acid
BMI: Body mass index
CKRT: Continuous kidney replacement therapy
CS: Cytosorb^®^
CVVHD(F): Continuous veno-venous hemodialysis/hemodiafiltration
ECMO: Extracorporeal membrane oxygenation
γ-GT: γ-Glutamyltransferase
GCA: Glycocholic acid
GCDCA: Glycochenodeoxycholic acid
GUDCA: Glycoursodeoxycholic acid
ICU: Intensive care unit
RR: Relative reduction
SAPS II: Simplified acute physiology score
TCA: Taurocholic acid
TCDCA: Taurochenodeoxycholic acid
TUDCA: Tauroursodeoxycholic acid
UDCA: Ursodeoxycholic acid
