# Supplementary material for: Extracorporeal adsorption of protective and toxic bile acids and bilirubin in patients with cholestatic liver dysfunction: a prospective study
Source: Ann Intensive Care. 2023 Nov 9;13:110. doi: 10.1186/s13613-023-01198-7 (PMC10635921; doi:10.1186/s13613-023-01198-7)
Supplement: Supplementary file 1 — Additional file 1. Table S1a: Total bilirubin concentration (mg/dL) pre- and post-Cytosorb® at different timepoints. Table S1b: Conjugated bilirubin concentration (mg/dL) pre- and post-Cytosorb® at different timepoints. Table S2a: GCA (µmol/L) concentration pre- and post-Cytosorb® at different timepoints. Table S2b: TCA (µmol/L) concentration pre- and post-Cytosorb® at different timepoints. Table S2c: GCDCA (µmol/L) concentration pre- and post-Cytosorb® at different timepoints. Table S2d: TCDCA (µmol/L) concentration pre- and post-Cytosorb® at different timepoints. Table S3a: UDCA (µmol/L) concentration pre- and post-Cytosorb® at different timepoints. Table S3b: GUDCA (µmol/L) concentration pre- and post-Cytosorb® at different timepoints. Table S3c: TUDCA (µmol/L) concentration pre- and post-Cytosorb® at different timepoints. Table S4: Blood concentration of different bile acids (µmol/L) before the start of Cytosorb® and after six and twelve hours after initiation. Figure S1: Relative reduction (%) of total bilirubin and conjugated bilirubin at different timepoints. [file 13613_2023_1198_MOESM1_ESM.docx]

**Supplemental File**

Table S1a: Total bilirubin concentration (mg/dL) pre- and post-Cytosorb^®^ at different timepoints

| Patient | D1 pre | D1 post | D2 pre | D2 post | D3 pre | D3 post | D4 pre | D4 post | D5 pre | D5 post |
| --- | --- | --- | --- | --- | --- | --- | --- | --- | --- | --- |
| 1 | 18.9 | 11.1 | 15.9 | 13.4 | 15.2 | 12.6 | 14.8 | 13.7 | 15.1 | 14.4 |
| 2 | 9.1 | 6.7 | 8.7 | 7.6 | 8.6 | 7.6 | 8.1 | 7.6 | 9.0 | 8.8 |
| 3 | 10.6 | 7.3 | 9.9 | 9.0 | 9.2 | 8.9 | 9.8 | 9.4 | 8.6 | 8.4 |
| 4 | 10.8 | 7.1 | 9.5 | 7.9 | 7.9 | 7.3 | 10.1 | 9.6 | 10.5 | 10.3 |
| 5 | 10.7 | 6.8 | 8.7 | 7.4 | 7.9 | 7.3 | 7.0 | 6.4 | 7.4 | 7.3 |
| 6 | 14.9 | 9.9 | 13.3 | 11.9 | 12.5 | 11.5 | 10.5 | 9.8 | 9.1 | 8.8 |
| 7 | 11.0 | 8.1 | 10.3 | 9.3 | 10.5 | 10.2 | 10.6 | 10.0 | 11.1 | 10.8 |
| 8 | 10.6 | 6.8 | 9.6 | 8.6 | 8.9 | 8.2 | 9.5 | 9.0 | 9.7 | 9.3 |
| 9 | 15.4 | 10.9 | 14.8 | 13.3 | 14.4 | 13.3 | 13.1 | 13.0 | 13.6 | 13.3 |
| 10 | 12.2 | 8.0 | 11.9 | 10.0 | 10.0 | 9.2 | 10.0 | 9.2 | 9.7 | 9.3 |
| 11 | 16.0 | 14.1 | 15.7 | 14.8 | 14.9 | 14.6 | 14.5 | 12.7 | 14.7 | 13.0 |
| 12 | 24.5 | 22.8 | 24.4 | 23.9 | 23.8 | 23.2 | 17.1 | 17.3 | 21.3 | 21.5 |
| 13 | 14.8 | 13.0 | 14.7 | 13.8 | 13.9 | 13.8 | 15.1 | 14.8 | 13.9 | 13.8 |
| 14 | 15.8 | 9.5 | 14.8 | 12.8 | 13.9 | 12.4 | 13.3 | 12.4 | 14.8 | 14.2 |
| 15 | 31.3 | 22.0 | 28.6 | 26.6 | 25.2 | 23.8 | 21.8 | 21.4 | 19.3 | 18.5 |
| 16 | 30.7 | 18.0 | 27.4 | 23.4 | 25.8 | 23.7 | 25.3 | 23.4 | 23.4 | 22.2 |
| 17 | 11.4 | 6.6 | 10.8 | 9.0 | 11.2 | 10.0 | 10.9 | 10.4 |  |  |
| 18 | 10.4 | 6.6 | 8.2 | 7.4 | 7.4 | 7.2 | 8.9 | 8.7 |  |  |
| 19 | 9.8 | 6.6 | 8.7 | 7.5 | 7.7 | 7.0 | 7.4 | 7.0 |  |  |
| 20 | 9.4 | 7.4 | 8.6 | 7.5 | 7.5 | 7.0 | 7.8 | 7.4 |  |  |

Note: D1: 10 min after initiation of Cytosorb^®^, D2: 1 h after initiation of Cytosorb^®^, D3: 3 h after initiation of Cytosorb^®^, D4: 6 h after initiation of Cytosorb^®^, D5: 12 h after initiation of Cytosorb^®^, pre: extracorporeal total bilirubin concentration pre-Cytosorb^®^, post: extracorporeal total bilirubin concentration post-Cytosorb^®^.

Table S1b: Conjugated bilirubin concentration (mg/dL) pre- and post-Cytosorb^®^ at different timepoints

| Patient | D1 pre | D1 post | D2 pre | D2 post | D3 pre | D3 post | D4 pre | D4 post | D5 pre | D5 post |
| --- | --- | --- | --- | --- | --- | --- | --- | --- | --- | --- |
| 1 | 15.6. | 9.2 | 13.4 | 11.3 | 13.0 | 10.9 | 12.6 | 12.0 | 13.0 | 12.3 |
| 2 | 8.0 | 5.9 | 7.5 | 6.6 | 7.4 | 6.7 | 7 | 6.6 | 7.8 | 7.6 |
| 3 | 9.9 | 6.9 | 9.5 | 8.4 | 8.7 | 8.3 | 9.1 | 8.8 | 8.1 | 7.9 |
| 4 | 2.9 | 1.9 | 2.4 | 2.0 | 1.8 | 1.7 | 2.1 | 2.0 | 2.1 | 2.1 |
| 5 | 10.3 | 6.7 | 8.5 | 7.3 | 7.7 | 7.1 | 6.9 | 6.4 | 7.3 | 7.2 |
| 6 | 12.1 | 8.5 | 11.0 | 10.2 | 10.3 | 10.0 | 9.0 | 8.5 | 7.6 | 7.6 |
| 7 | 9.1 | 6.7 | 8.6 | 7.7 | 8.7 | 8.5 | 8.9 | 8.4 | 9.6 | 9.5 |
| 8 | 10.1 | 6.7 | 9.3 | 8.4 | 8.4 | 7.8 | 8.9 | 8.5 | 9.0 | 8.8 |
| 9 | 14.0 | 10.3 | 13.5 | 12.3 | 13.1 | 12.3 | 12.2 | 11.9 | 12.5 | 12.3 |
| 10 | 11.2 | 7.6 | 11.0 | 9.3 | 9.6 | 8.7 | 9.4 | 8.6 | 9.1 | 8.7 |
| 11 | 12.9 | 11.6 | 12.7 | 12.2 | 12.2 | 11.9 | 11.7 | 10.6 | 11.9 | 10.7 |
| 12 | 10.7 | 10.0 | 10.4 | 10.2 | 9.9 | 9.7 | 7.3 | 7.6 | 9.0 | 9.0 |
| 13 | 11.3 | 10.0 | 11.2 | 10.6 | 10.7 | 10.6 | 11.6 | 11.2 | 10.9 | 10.8 |
| 14 | 13.6 | 8.7 | 13.1 | 11.2 | 12.4 | 11.1 | 11.7 | 11.0 | 12.9 | 12.3 |
| 15 | 25.5 | 17.0 | 23.9 | 22.2 | 21.5 | 19.9 | 18.6 | 16.9 | 15.0 | 14.9 |
| 16 | 25,4 | 14.7 | 23.2 | 20.1 | 21.9 | 20.2 | 21.6 | 20.0 | 20.2 | 19.1 |
| 17 | 10.2 | 6.0 | 9.8 | 8.3 | 10.0 | 9.0 | 10.0 | 9.4 |  |  |
| 18 | 4.3 | 2.3 | 3.1 | 2.8 | 2.4 | 2.4 | 2.5 | 2.5 |  |  |
| 19 | 8.5 | 5.9 | 7.4 | 6.6 | 6.5 | 6.0 | 6.4 | 6.0 |  |  |
| 20 | 8.3 | 6.6 | 7.5 | 6.6 | 6.5 | 6.1 | 6.9 | 6.7 |  |  |

Note: D1: 10 min after initiation of Cytosorb^®^, D2: 1 h after initiation of Cytosorb^®^, D3: 3 h after initiation of Cytosorb^®^, D4: 6 h after initiation of Cytosorb^®^, D5: 12 h after initiation of Cytosorb^®^, pre: extracorporeal conjugated bilirubin concentration pre-Cytosorb^®^, post: extracorporeal conjugated bilirubin concentration post-Cytosorb^®^.

Table S2a: GCA (µmol/L) concentration pre- and post-Cytosorb^®^ at different timepoints

| Patient | D1 pre | D1 post | D2 pre | D2 post | D3 pre | D3 post | D4 pre | D4 post | D5 pre | D5 post |
| --- | --- | --- | --- | --- | --- | --- | --- | --- | --- | --- |
| 1 | 21.4 | 0.1 | 16.1 | 1.6 | 14.8 | 3.3 | 11.9 | 7.0 | 10.5 | 5.5 |
| 2 | 2.8 | 0.0 | 2.5 | 0.3 | 2.1 | 0.6 | 2.5 | 1.0 | 3.0 | 1.7 |
| 3 | 5.2 | 0.0 | 3.7 | 0.7 | 4.2 | 1.7 | 2.4 | 1.5 | 3.3 | 2.0 |
| 4 | 7.1 | 0.1 | 5.9 | 0.8 | 4.0 | 1.6 | 4.1 | 3.3 | 3.0 | 3.0 |
| 5 | 6.5 | 0.1 | 5.1 | 0.5 | 4.6 | 1.6 | 3.7 | 1.8 | 4.3 | 2.6 |
| 6 | 2.3 | 0.0 | 1.9 | 0.1 | 1.8 | 0.4 | 1.5 | 0.5 | 0.9 | 0.7 |
| 7 | 1.6 | 0.0 | 1.3 | 0.2 | 1.6 | 0.6 | 2.2 | 1.1 | 2.2 | 1.8 |
| 8 | 3.6 | 0.1 | 3.0 | 0.7 | 2.0 | 0.9 | 1.6 | 1.0 | 1.6 | 1.4 |
| 9 | 5.0 | 0.2 | 4.7 | 1.9 | 4.3 | 2.5 | 3.6 | 2.6 | 3.4 | 3.0 |
| 10 | 10.0 | 0.3 | 9.2 | 1.6 | 6.7 | 3.3 | 6.0 | 3.8 | 5.4 | 4.4 |
| 11 | 3.1 | 0.3 | 3.3 | 1.5 | 26.2 | 9.8 | 8.6 | 5.8 | 4.4 | 4.0 |
| 12 | 2.3 | 0.8 | 2.4 | 1.2 | 2.1 | 1.6 | 1.8 | 1.5 | 2.1 | 1.7 |
| 13 | 4.2 | 0.6 | 3.6 | 2.0 | 2.8 | 2.5 | 6.8 | 4.5 | 7.3 | 6.3 |
| 14 | 51.2 | 2.2 | 40.0 | 12.4 | 34.1 | 21.7 | 29.5 | 20.5 | 25.9 | 24.1 |
| 15 | 17.8 | 0.2 | 14.9 | 4.9 | 14.9 | 6.3 | 10.2 | 7.2 | 10.6 | 8.3 |
| 16 | 10.8 | 0.2 | 10.2 | 1.4 | 8.0 | 3.5 | 7.0 | 4.7 | 10.8 | 8.5 |
| 17 | 9.0 | 0.1 | 7.7 | 1.1 | 7.4 | 3.0 | 9.8 | 5.6 |  |  |
| 18 | 1.6 | 0.0 | 0.9 | 0.2 | 0.7 | 0.4 | 0.6 | 0.4 |  |  |
| 19 | 29.0 | 0.2 | 24.3 | 2.2 | 16.7 | 5.0 | 12.9 | 6.2 |  |  |
| 20 | 14.3 | 1.2 | 12.8 | 5.4 | 11.5 | 7.4 | 10.9 | 9.5 |  |  |

Note: GCA: glycocholic acid, D1: 10 min after initiation of Cytosorb^®^, D2: 1 h after initiation of Cytosorb^®^, D3: 3 h after initiation of Cytosorb^®^, D4: 6 h after initiation of Cytosorb^®^, D5: 12 h after initiation of Cytosorb^®^, pre: extracorporeal GCA concentration pre-Cytosorb^®^, post: extracorporeal GCA concentration post-Cytosorb^®^.

Table S2b: TCA (µmol/L) concentration pre- and post-Cytosorb^®^ at different timepoints

| Patient | D1 pre | D1 post | D2 pre | D2 post | D3 pre | D3 post | D4 pre | D4 post | D5 pre | D5 post |
| --- | --- | --- | --- | --- | --- | --- | --- | --- | --- | --- |
| 1 | 2.3 | 0.0 | 1.8 | 0.2 | 1.7 | 0.4 | 1.5 | 0.9 | 1.4 | 0.7 |
| 2 | 0.6 | 0.0 | 0.5 | 0.1 | 0.5 | 0.1 | 0.5 | 0.2 | 0.5 | 0.3 |
| 3 | 3.1 | 0.1 | 2.5 | 0.7 | 3.0 | 1.4 | 2.0 | 1.3 | 2.6 | 1.8 |
| 4 | 6.4 | 0.1 | 5.4 | 0.8 | 3.9 | 1.5 | 3.9 | 2.8 | 2.8 | 2.7 |
| 5 | 2.5 | 0.2 | 2.2 | 0.4 | 1.9 | 0.8 | 1.7 | 0.9 | 1.8 | 1.1 |
| 6 | 0.9 | 0.0 | 1.0 | 0.1 | 1.0 | 0.3 | 0.9 | 0.3 | 0.6 | 0.5 |
| 7 | 2.3 | 0.1 | 1.7 | 0.4 | 2.0 | 1.0 | 2.5 | 1.3 | 2.5 | 2.2 |
| 8 | 4.3 | 0.2 | 3.9 | 1.1 | 2.5 | 1.3 | 2.2 | 1.4 | 2.2 | 1.9 |
| 9 | 7.8 | 0.5 | 7.7 | 3.2 | 7.3 | 4.4 | 5.6 | 4.3 | 5.6 | 4.7 |
| 10 | 3.1 | 0.2 | 3.0 | 0.7 | 2.1 | 1.2 | 2.0 | 1.3 | 1.9 | 0.7 |
| 11 | 0.6 | 0.1 | 0.7 | 0.3 | 1.2 | 0.7 | 0.9 | 0.6 | 0.5 | 0.5 |
| 12 | 1.2 | 0.4 | 1.0 | 0.6 | 1.0 | 0.8 | 0.9 | 0.8 | 1.3 | 1.2 |
| 13 | 0.4 | 0.1 | 0.4 | 0.3 | 0.3 | 0.3 | 0.4 | 0.3 | 0.3 | 0.3 |
| 14 | 9.2 | 0.6 | 7.5 | 2.7 | 6.3 | 4.4 | 4.7 | 3.5 | 4.8 | 4.3 |
| 15 | 14.5 | 0.2 | 13.3 | 4.6 | 10.9 | 4.8 | 8.6 | 5.7 | 5.2 | 4.7 |
| 16 | 9.8 | 0.4 | 10.3 | 2.8 | 8.0 | 3.8 | 7.4 | 5.0 | 7.5 | 6.2 |
| 17 | 4.1 | 0.1 | 3.7 | 0.7 | 3.7 | 1.6 | 3.6 | 2.0 |  |  |
| 18 | 0.6 | 0.0 | 0.3 | 0.1 | 0.3 | 0.2 | 0.2 | 0.2 |  |  |
| 19 | 4.6 | 0.2 | 4.6 | 0.6 | 3.0 | 1.0 | 2.4 | 1.3 |  |  |
| 20 | 2.2 | 0.2 | 2.0 | 0.9 | 1.8 | 1.1 | 2.1 | 1.7 |  |  |

Note: TCA: taurocholic acid, D1: 10 min after initiation of Cytosorb^®^, D2: 1 h after initiation of Cytosorb^®^, D3: 3 h after initiation of Cytosorb^®^, D4: 6 h after initiation of Cytosorb^®^, D5: 12 h after initiation of Cytosorb^®^, pre: extracorporeal TCA concentration pre-Cytosorb^®^, post: extracorporeal TCA concentration post-Cytosorb^®^.

Table S2c: GCDCA (µmol/L) concentration pre- and post-Cytosorb^®^ at different timepoints

| Patient | D1 pre | D1 post | D2 pre | D2 post | D3 pre | D3 post | D4 pre | D4 post | D5 pre | D5 post |
| --- | --- | --- | --- | --- | --- | --- | --- | --- | --- | --- |
| 1 | 28.4 | 3.7 | 23.4 | 15.2 | 21.1 | 14.0 | 19.5 | 15.1 | 17.8 | 14.3 |
| 2 | 2.7 | 0.3 | 2.7 | 1.5 | 2.4 | 1.9 | 2.4 | 1.9 | 2.1 | 1.7 |
| 3 | 9.0 | 0.8 | 7.6 | 4.4 | 9.0 | 6.1 | 7.8 | 7.0 | 9.4 | 7.6 |
| 4 | 14.2 | 3.5 | 11.3 | 8.1 | 10.4 | 8.1 | 10.8 | 9.6 | 9.0 | 9.7 |
| 5 | 11.8 | 0.8 | 10.2 | 5.1 | 8.3 | 6.4 | 6.9 | 5.4 | 10.0 | 7.3 |
| 6 | 11.8 | 1.9 | 9.9 | 6.7 | 9.2 | 7.7 | 7.6 | 6.2 | 5.5 | 5.6 |
| 7 | 5.5 | 2.1 | 5.8 | 5.0 | 8.1 | 8.5 | 8.7 | 7.8 | 6.5 | 7.3 |
| 8 | 3.1 | 0.4 | 3.0 | 1.9 | 2.4 | 1.8 | 2.2 | 1.8 | 2.2 | 1.9 |
| 9 | 3.5 | 1.0 | 3.9 | 2.8 | 3.4 | 3.0 | 3.5 | 3.0 | 3.2 | 3.0 |
| 10 | 6.9 | 1.0 | 6.4 | 3.3 | 5.0 | 4.2 | 4.7 | 4.2 | 3.4 | 4.2 |
| 11 | 6.1 | 4.3 | 6.8 | 6.2 | 10.0 | 9.2 | 10.1 | 7.7 | 8.2 | 7.9 |
| 12 | 41.0 | 31.1 | 39.6 | 40.8 | 40.0 | 39.4 | 30.0 | 31.0 | 35.7 | 35.5 |
| 13 | 23.7 | 16.5 | 20.5 | 16.8 | 18.0 | 19.0 | 20.1 | 19.5 | 16,4 | 15.7 |
| 14 | 16.7 | 2.8 | 16.2 | 10.0 | 14.6 | 10.8 | 12.0 | 9.0 | 9.8 | 9.4 |
| 15 | 23.2 | 4.6 | 19.4 | 15.5 | 18.4 | 16.5 | 17.1 | 15.5 | 14.1 | 12.0 |
| 16 | 6.5 | 0.9 | 5.6 | 3.4 | 5.5 | 4.5 | 4.7 | 4.6 | 4.3 | 4.1 |
| 17 | 5.5 | 0.4 | 5.2 | 2.9 | 6.0 | 4.7 | 6.0 | 4.7 |  |  |
| 18 | 7.3 | 1.1 | 4.6 | 3.7 | 4.6 | 4.1 | 4.0 | 3.9 |  |  |
| 19 | 38.8 | 7.0 | 29.3 | 16.8 | 29.6 | 21.4 | 27.5 | 22.8 |  |  |
| 20 | 5.0 | 2.1 | 4.1 | 3.4 | 4.4 | 3.8 | 4.6 | 4.2 |  |  |

Note: GCDCA: glycochenodeoxycholic acid, D1: 10 min after initiation of Cytosorb^®^, D2: 1 h after initiation of Cytosorb^®^, D3: 3 h after initiation of Cytosorb^®^, D4: 6 h after initiation of Cytosorb^®^, D5: 12 h after initiation of Cytosorb^®^, pre: extracorporeal GCDCA concentration pre-Cytosorb^®^, post: extracorporeal GCDCA concentration post-Cytosorb^®^.

Table S2d: TCDCA (µmol/L) concentration pre- and post-Cytosorb^®^ at different timepoints

| Patient | D1 pre | D1 post | D2 pre | D2 post | D3 pre | D3 post | D4 pre | D4 post | D5 pre | D5 post |
| --- | --- | --- | --- | --- | --- | --- | --- | --- | --- | --- |
| 1 | 5.5 | 0.3 | 4.2 | 2.1 | 4.1 | 2.4 | 3.7 | 3.3 | 3.3 | 2.7 |
| 2 | 0.4 | 0.0 | 0.4 | 0.2 | 0.3 | 0.2 | 0.3 | 0.3 | 0.3 | 0.2 |
| 3 | 3.8 | 0.3 | 3.5 | 2.0 | 4.6 | 3.4 | 3.8 | 3.1 | 5.2 | 3.8 |
| 4 | 7.0 | 1.4 | 5.5 | 3.9 | 4.4 | 3.6 | 5.1 | 4.7 | 4.3 | 4.7 |
| 5 | 3.1 | 0.2 | 3.0 | 1.3 | 2.5 | 1.7 | 2.0 | 1.5 | 2.8 | 2.1 |
| 6 | 5.1 | 0.5 | 5.0 | 2.1 | 4.9 | 3.1 | 4.4 | 3.1 | 3.0 | 2.9 |
| 7 | 6.2 | 1.8 | 5.5 | 4.6 | 6.1 | 5.9 | 6.4 | 5.2 | 4.8 | 5.1 |
| 8 | 3.5 | 0.5 | 3.4 | 2.2 | 2.4 | 2.0 | 2.5 | 2.1 | 2.6 | 2.3 |
| 9 | 4.1 | 0.9 | 4.6 | 3.0 | 4.4 | 3.5 | 3.7 | 3.3 | 3.5 | 3.1 |
| 10 | 1.4 | 0.2 | 1.5 | 0.8 | 1.2 | 0.9 | 1.1 | 0.9 | 1.0 | 0.9 |
| 11 | 2.2 | 1.5 | 2.7 | 2.4 | 3.7 | 3.4 | 3.3 | 2.7 | 2.2 | 2.0 |
| 12 | 14.4 | 11.6 | 14.7 | 13.2 | 13.9 | 13.1 | 11.3 | 11.5 | 16.5 | 15.4 |
| 13 | 4.1 | 3.0 | 4.0 | 3.5 | 3.5 | 3.7 | 3.8 | 3.6 | 3.2 | 2.9 |
| 14 | 3.2 | 0.7 | 3.3 | 2.0 | 2.9 | 2.4 | 2.4 | 2.0 | 2.3 | 2.2 |
| 15 | 18.6 | 2.4 | 16.5 | 14.0 | 16.4 | 13.1 | 14.2 | 13.5 | 9.6 | 8.8 |
| 16 | 12.3 | 1.6 | 11.3 | 7.4 | 11.4 | 8.6 | 9.2 | 9.1 | 9.4 | 8.9 |
| 17 | 3.4 | 0.2 | 3.5 | 1.9 | 4.0 | 2.9 | 3.6 | 3.1 |  |  |
| 18 | 1.1 | 0.1 | 0.7 | 0.6 | 0.7 | 0.6 | 0.6 | 0.5 |  |  |
| 19 | 10.7 | 0.9 | 9.0 | 4.0 | 8.3 | 5.6 | 7.2 | 6.3 |  |  |
| 20 | 0.8 | 0.3 | 0.7 | 0.6 | 0.8 | 0.6 | 0.8 | 0.7 |  |  |

Note: TCDCA: taurochenodeoxycholic acid, D1: 10 min after initiation of Cytosorb^®^, D2: 1 h after initiation of Cytosorb^®^, D3: 3 h after initiation of Cytosorb^®^, D4: 6 h after initiation of Cytosorb^®^, D5: 12 h after initiation of Cytosorb^®^, pre: extracorporeal TCDCA concentration pre-Cytosorb^®^, post: extracorporeal TCDCA concentration post-Cytosorb^®^.

Table S3a: UDCA (µmol/L) concentration pre- and post-Cytosorb^®^ at different timepoints

| Patient | D1 pre | D1 post | D2 pre | D2 post | D3 pre | D3 post | D4 pre | D4 post | D5 pre | D5 post |
| --- | --- | --- | --- | --- | --- | --- | --- | --- | --- | --- |
| 1 | 10.1 | 1.5 | 10.5 | 6.1 | 21.0 | 11.5 | 10.7 | 10.3 | 4.5 | 4.8 |
| 2 | 1.7 | 0.2 | 1.4 | 0.9 | 5.7 | 3.5 | 1.2 | 1.0 | 1.2 | 1.0 |
| 3 | 0.3 | 0.0 | 0.3 | 0.2 | 0.2 | 0.2 | 1.2 | 0.8 | 0.7 | 0.6 |
| 4 | 9.6 | 3.0 | 8.6 | 6.2 | 11.4 | 8.5 | 15.9 | 14.2 | 14.3 | 14.5 |
| 5 | 1.6 | 0.1 | 1.2 | 0.5 | 0.4 | 0.4 | 0.3 | 0.3 | 3.7 | 2.7 |
| 6 | 1.4 | 0.3 | 1.0 | 0.8 | 0.7 | 0.6 | 4.3 | 2.7 | 1.2 | 1.2 |
| 7 | 0.7 | 0.2 | 0.4 | 0.3 | 0.3 | 0.3 | 0.2 | 0.2 | 0.2 | 0.2 |
| 8 | 0.3 | 0.0 | 0.2 | 0.1 | 0.5 | 0.3 | 3.4 | 1.4 | 0.4 | 0.4 |
| 9 | 0.5 | 0.1 | 0.4 | 0.3 | 0.7 | 0.6 | 0.4 | 0.4 | 1.1 | 1.0 |
| 10 | 0.7 | 0.1 | 1.3 | 0.6 | 0.8 | 0.7 | 0.9 | 0.7 | 0.7 | 0.7 |
| 11 | 0.4 | 0.3 | 6.9 | 5.7 | 9.7 | 8.7 | 9.2 | 7.4 | 2.3 | 2.3 |
| 12 | 20.4 | 16.4 | 22.3 | 20.1 | 25.2 | 23.4 | 17.4 | 17.0 | 32.6 | 29.1 |
| 13 | 8.9 | 6.6 | 20.8 | 17.1 | 31.3 | 33.5 | 26.0 | 23.7 | 14.4 | 14.1 |
| 14 | 0.5 | 0.2 | 0.4 | 0.2 | 0.2 | 0.1 | 0.1 | 0.1 | 0.0 | 0.0 |
| 15 | 2.2 | 0.5 | 1.9 | 1.4 | 10.6 | 9.2 | 13.3 | 12.3 | 1.7 | 1.9 |
| 16 | 0.2 | 0.0 | 0.1 | 0.1 | 0.1 | 0.1 | 0.1 | 0.1 | 0.1 | 0.1 |
| 17 | 0.3 | 0.0 | 0.4 | 0.2 | 0.3 | 0.2 | 0.2 | 0.2 |  |  |
| 18 | 7.2 | 2.5 | 5.0 | 4.2 | 5.0 | 4.6 | 3.4 | 4.4 |  |  |
| 19 | 4.7 | 1.0 | 4.7 | 2.8 | 6.5 | 4.8 | 7.8 | 7.3 |  |  |
| 20 | 0.1 | 0.0 | 0.1 | 0.1 | 0.1 | 0.1 | 0.1 | 0.1 |  |  |

Note: UDCA: ursodeoxycholic acid, D1: 10 min after initiation of Cytosorb^®^, D2: 1 h after initiation of Cytosorb^®^, D3: 3 h after initiation of Cytosorb^®^, D4: 6 h after initiation of Cytosorb^®^, D5: 12 h after initiation of Cytosorb^®^, pre: extracorporeal UDCA concentration pre-Cytosorb^®^, post: extracorporeal UDCA concentration post-Cytosorb^®^.

Table S3b: GUDCA (µmol/L) concentration pre- and post-Cytosorb^®^ at different timepoints

| Patient | D1 pre | D1 post | D2 pre | D2 post | D3 pre | D3 post | D4 pre | D4 post | D5 pre | D5 post |
| --- | --- | --- | --- | --- | --- | --- | --- | --- | --- | --- |
| 1 | 124.3 | 9.5 | 98.1 | 56.1 | 103.9 | 66.4 | 110.2 | 103.2 | 98.3 | 84.9 |
| 2 | 16.8 | 1.3 | 12.2 | 8.6 | 11.1 | 7.1 | 9.6 | 8.1 | 6.0 | 5.7 |
| 3 | 14.8 | 0.5 | 11.5 | 6.0 | 12.4 | 7.7 | 13.0 | 12.2 | 14.7 | 11.4 |
| 4 | 57.7 | 21.7 | 47.5 | 36.9 | 41.0 | 35.2 | 47.0 | 42.4 | 44.8 | 43.8 |
| 5 | 19.5 | 0.7 | 16.3 | 5.2 | 13.0 | 8.5 | 9.8 | 6.8 | 14.3 | 11.6 |
| 6 | 90.6 | 14.7 | 73.1 | 47.8 | 66.4 | 47.6 | 49.2 | 44.6 | 35.1 | 34.3 |
| 7 | 1.1 | 0.4 | 1.5 | 1.2 | 1.8 | 1.7 | 2.4 | 2.0 | 1.9 | 1.9 |
| 8 | 6.9 | 0.5 | 5.9 | 3.0 | 4.9 | 3.9 | 5.8 | 4.9 | 8.0 | 7.4 |
| 9 | 5.7 | 0.9 | 5.3 | 3.4 | 5.2 | 3.9 | 4.8 | 4.2 | 4.9 | 4.4 |
| 10 | 15.2 | 1.2 | 13.5 | 6.3 | 12.9 | 8.6 | 9.7 | 7.9 | 12.5 | 10.3 |
| 11 | 8.1 | 7.0 | 16.9 | 13.5 | 35.0 | 32.7 | 55.1 | 51.1 | 45.3 | 39.6 |
| 12 | 142.9 | 114.6 | 140.2 | 137.9 | 137.7 | 137.2 | 106.1 | 112.0 | 144.5 | 153.1 |
| 13 | 66.7 | 50.7 | 64.3 | 57.4 | 76.4 | 77.9 | 107.4 | 101.0 | 102.3 | 108.7 |
| 14 | 29.6 | 1.9 | 25.1 | 10.4 | 18.2 | 12.9 | 12.1 | 10.7 | 7.2 | 7.3 |
| 15 | 117.2 | 24.7 | 83.7 | 76.7 | 105.9 | 87.5 | 110.6 | 103.5 | 76.0 | 73.1 |
| 16 | 4.9 | 0.4 | 3.9 | 2.4 | 4.1 | 3.2 | 3.2 | 3.0 | 2.5 | 2.5 |
| 17 | 3.0 | 0.1 | 3.2 | 1.5 | 4.0 | 3.1 | 3.0 | 2.3 |  |  |
| 18 | 7.5 | 1.4 | 5.0 | 4.4 | 4.6 | 4.3 | 4.0 | 3.7 |  |  |
| 19 | 117.6 | 19.3 | 96.0 | 50.1 | 93.3 | 72.0 | 83.9 | 73.3 |  |  |
| 20 | 1.2 | 0.5 | 1.1 | 0.9 | 1.2 | 1.1 | 1.1 | 1.0 |  |  |

Note: GUDCA: glycoursodeoxycholic acid, D1: 10 min after initiation of Cytosorb^®^, D2: 1 h after initiation of Cytosorb^®^, D3: 3 h after initiation of Cytosorb^®^, D4: 6 h after initiation of Cytosorb^®^, D5: 12 h after initiation of Cytosorb^®^, pre: extracorporeal GUDCA concentration pre-Cytosorb^®^, post: extracorporeal GUDCA concentration post-Cytosorb^®^.

Table S3c: TUDCA (µmol/L) concentration pre- and post-Cytosorb^®^ at different timepoints

| Patient | D1 pre | D1 post | D2 pre | D2 post | D3 pre | D3 post | D4 pre | D4 post | D5 pre | D5 post |
| --- | --- | --- | --- | --- | --- | --- | --- | --- | --- | --- |
| 1 | 18.8 | 0.8 | 14.3 | 6.7 | 15.4 | 8.8 | 14.9 | 13.1 | 12.7 | 11.1 |
| 2 | 1.3 | 0.0 | 1.0 | 0.5 | 0.9 | 0.5 | 0.7 | 0.6 | 0.5 | 0.4 |
| 3 | 4.3 | 0.1 | 3.7 | 1.5 | 4.9 | 2.8 | 4.2 | 3.2 | 5.6 | 4.2 |
| 4 | 14.4 | 3.8 | 12.3 | 11.5 | 10.3 | 8.9 | 11.8 | 11.2 | 10.0 | 11.5 |
| 5 | 3.1 | 0.1 | 2.7 | 0.7 | 2.1 | 1.2 | 1.5 | 1.0 | 3.1 | 1.9 |
| 6 | 29.6 | 2.4 | 23.4 | 17.1 | 19.5 | 15.3 | 18.7 | 13.5 | 11.0 | 11.6 |
| 7 | 1.6 | 0.4 | 2.0 | 1.6 | 2.2 | 2.0 | 2.3 | 2.0 | 1.5 | 1.7 |
| 8 | 5.0 | 0.2 | 4.5 | 2.0 | 3.8 | 2.8 | 6.4 | 4.7 | 7.2 | 6.4 |
| 9 | 5.2 | 0.5 | 4.7 | 2.6 | 4.6 | 3.2 | 4.0 | 3.5 | 4.0 | 3.4 |
| 10 | 1.7 | 0.1 | 1.6 | 0.5 | 1.3 | 0.9 | 1.0 | 0.8 | 1.2 | 1.0 |
| 11 | 2.0 | 1.5 | 3.7 | 3.4 | 6.7 | 6.1 | 6.2 | 5.3 | 3.5 | 3.3 |
| 12 | 15.5 | 12.5 | 16.8 | 15.5 | 17.4 | 15.8 | 14.8 | 15.4 | 26.3 | 26.2 |
| 13 | 8.0 | 5.9 | 8.2 | 7.3 | 8.8 | 9.6 | 11.8 | 10.8 | 10.8 | 10.8 |
| 14 | 3.3 | 0.1 | 3.0 | 1.1 | 2.2 | 1.5 | 1.4 | 1.1 | 0.7 | 0.7 |
| 15 | 60.1 | 6.6 | 42.3 | 38.0 | 55.2 | 43.6 | 57.2 | 52.5 | 31.6 | 30.7 |
| 16 | 4.5 | 0.3 | 4.0 | 2.1 | 3.6 | 2.6 | 2.9 | 2.7 | 2.3 | 2.3 |
| 17 | 1.5 | 0.0 | 1.7 | 0.7 | 2.0 | 1.3 | 1.3 | 1.1 |  |  |
| 18 | 0.6 | 0.1 | 0.4 | 0.3 | 0.3 | 0.3 | 0.2 | 0.2 |  |  |
| 19 | 26.3 | 1.9 | 20.7 | 8.6 | 18.6 | 12.9 | 18.1 | 13.6 |  |  |
| 20 | 0.2 | 0.1 | 0.2 | 0.1 | 0.2 | 0.2 | 0.2 | 0.2 |  |  |

Note: TUDCA: tauroursodeoxycholic acid, D1: 10 min after initiation of Cytosorb^®^, D2: 1 h after initiation of Cytosorb^®^, D3: 3 h after initiation of Cytosorb^®^, D4: 6 h after initiation of Cytosorb^®^, D5: 12 h after initiation of Cytosorb^®^, pre: extracorporeal TUDCA concentration pre-Cytosorb^®^, post: extracorporeal TUDCA concentration post-Cytosorb^®^.

**Table S4:** Blood concentration of different bile acids (µmol/L) before the start of Cytosorb^®^ and after 6 and 12 h after initiation

|  | **Before therapy start** | **Six hours after initiation** | **Twelve hours after initiation** |
| --- | --- | --- | --- |
| GCA median (IQR) | 6.8  (4.7, 12.3) | 4.2  (3.0, 8.8) | 4.5  (3.2, 9.1) |
| TCA median (IQR) | 3.4  (2.0, 7.6) | 2.3  (1.1, 4.3) | 2.5  (1.0, 3.8) |
| GCDCA median (IQR) | 12.5  (7.5, 19.7) | 8.6  (4.9, 12.5) | 9.0  (5.0, 10.1) |
| TCDCA median (IQR) | 5.4  (3.5, 8.8) | 3.6  (2.5, 5.1) | 3.3  (2.6, 4.6) |
| UDCA median (IQR) | 1.2  (0.5, 3.1) | 2.3  (0.3, 10.4) | 1.2  (0.6, 3.8) |
| GUDCA median (IQR) | 21.1  (10.8, 93.4) | 14.0  (8.0, 67.4) | 15.1  (6.9, 57.1) |
| TUDCA median (IQR) | 6.0  (3.1, 15.2) | 5.8  (2.1, 11.5) | 4.9  (2.5, 11.5) |

Note: GCA: glycocholic acid, TCA: taurocholic acid, GCDCA: glycochenodeoxycholic acid, TCDCA: taurochenodeoxycholic acid, UDCA: ursodeoxycholic acid, GUDCA: glycoursodeoxycholic acid, TUDCA: tauroursodeoxycholic acid

**Figure S1:** Relative reduction (%) of total bilirubin and conjugated bilirubin at different timepoints


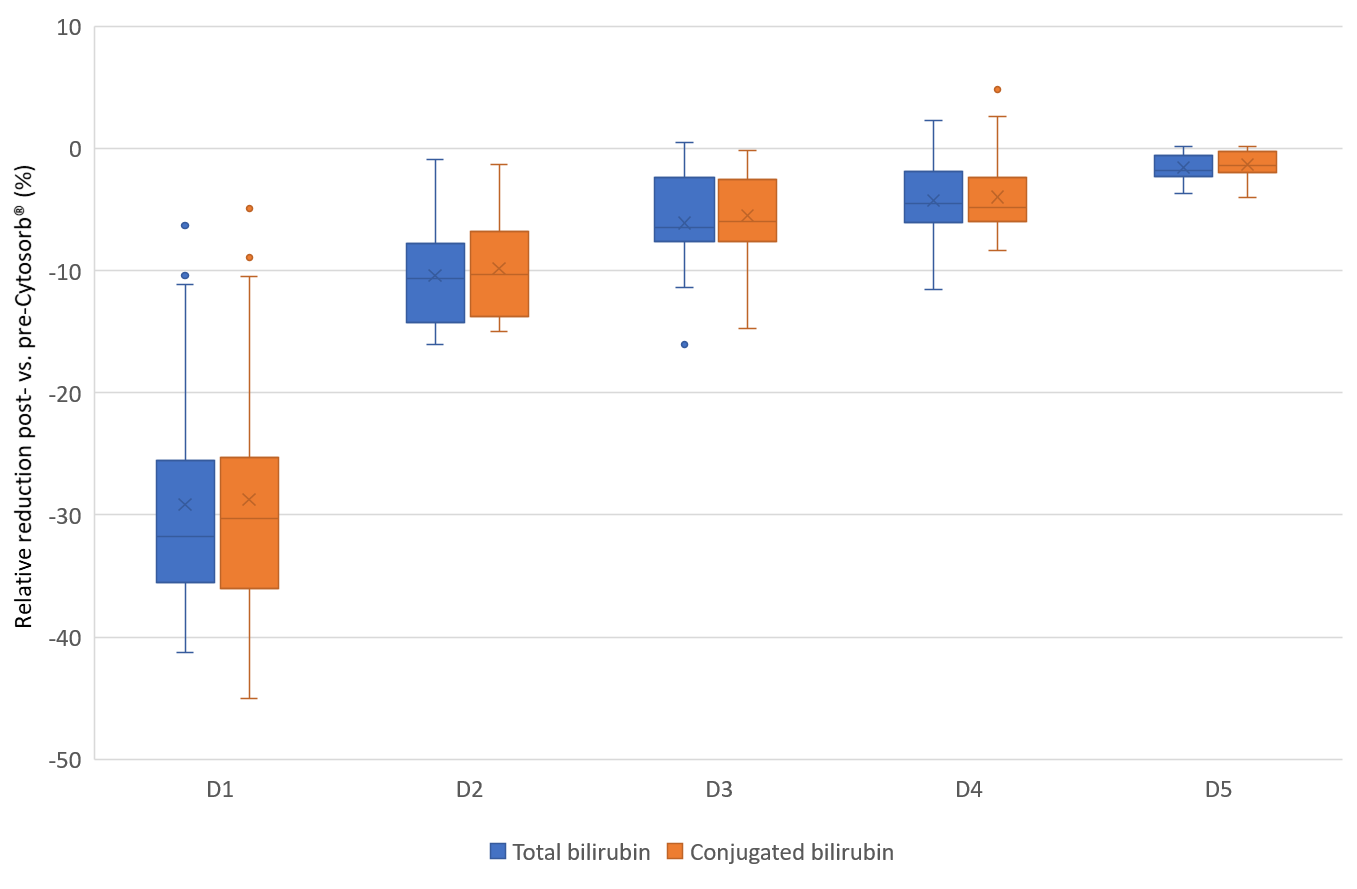


Note: D1: 10 min after initiation of Cytosorb^®^, D2: 1 h after initiation of Cytosorb^®^, D3: 3 h after initiation of Cytosorb^®^, D4: 6 h after initiation of Cytosorb^®^, D5: 12 h after initiation of Cytosorb^®^, blue boxplots represent the relative reduction of total bilirubin and orange ones of conjugated bilirubin. The boxes of the boxplots represent the interquartile range (IQR) and the line the median. Whiskers were limited to 1.5 times the IQR. The cross represents the mean.
